# Longitudinal associations between social media use, mental well-being and structural brain development across adolescence^☆^

**DOI:** 10.1016/j.dcn.2022.101088

**Published:** 2022-02-19

**Authors:** Michelle Achterberg, Andrik Becht, Renske van der Cruijsen, Ilse H. van de Groep, Jochem P. Spaans, Eduard Klapwijk, Eveline A. Crone

**Affiliations:** a Erasmus University Rotterdam, The Netherlands; b Leiden University, The Netherlands; c Utrecht University, The Netherlands; d Amsterdam University Medical Center, The Netherlands

**Keywords:** Social media, Adolescence, Mental well-being, Cortical thickness, Surface area

## Abstract

Youth of today grow up in a digital social world but the effects on well-being and brain development remain debated. This study tracked longitudinal associations between structural brain development, social media use and mental well-being.

The study demonstrated two pathways of heterogeneity in brain development. First, adolescents who used social media more than their peers showed higher baseline cortical thickness in lateral prefrontal cortex (PFC) and medial PFC; and stronger decreases in the lateral PFC and temporal parietal junction. In contrast, adolescents with lower mental well-being showed lower baseline levels of surface area in the medial PFC and posterior superior temporal sulcus relative to their peers. Whereas the associations between structural brain development and well-being remained significant after correction for multiple testing, the results for social media use did not survive FDR correction.

These findings demonstrate that although social media use and mental well-being were both associated with differential trajectories of brain development, the associations we report are distinct. These results show a nuanced perspective on the presumed relations between social media use and well-being and provide a starting point to further examine neural mechanisms that could explain which adolescents thrive by social media and which might be harmed.

## Highlights

• Longitudinal study with three time points across adolescence on MPFC, TPJ, pSTS and LPFC development.
• Cortical thickness development differed between high vs low social media users.
• Surface area development differed between low vs high mental well-being subgroups.
• Associations with well-being remained significant after stringent FDR correction.
• Social media and well-being are distinctly associated with structural brain development.

## 1 Introduction

Ever since the launch of *MySpace* in 2003 there has been an incredible rise of social media platforms (Ortiz-Ospina, 2019). As with most new technologies, social media has been particularly attractive to adolescents. Social media provides immediate and often rewarding feedback and easily connects adolescents with their friends (Odgers and Robb, 2020). While friendships play an important role across all ages, the need to socially connect to peers is a central aspect of young peoples’ lives (Denworth, 2020). Adolescence can be viewed as a period for social reorienting, as adolescents’ motivation to engage with peers and to build their own social networks naturally increases (Larson and Richards, 1991; Steinberg and Morris, 2001; Crone and Dahl, 2012). Adolescents’ specific biological and psychological changes trigger a heightened sensitivity to social stimuli and increased need for interaction with peers (Blakemore, 2008). As adolescents spend more time with peers and experience this heightened sensitivity to peer acceptance and peer rejection (Blakemore and Mills, 2014), they also have been shown to experience more frequent peer-based stressors, and react emotionally to peer-stressors, in comparison to children (Brown and Larson, 2009). Thus, during this phase of development, adolescents are particularly sensitive to their social environment (Somerville et al., 2010). Currently, it remains unclear how the social connectedness though social media is associated with brain development throughout adolescence, and vice versa (Crone and Konijn, 2018). Intense social experiences might influence the development of the adolescent brain, whereas at the same time, adolescents may differ in the extent to which they seek out social experiences. Longitudinal research across adolescence is crucial to examine how social media use and brain development are associated over time. The current study aimed to shed light on individual differences in longitudinal associations between social media use and structural brain development across adolescence.

Heightened sensitivity to peer feedback during adolescence has left caregivers worried about social media platforms and social media use among adolescents (Martinotti et al., 2011). While some researchers have suggested that social media use is related to an increase in depressive symptoms and suicide-related outcomes (Twenge et al., 2018), most scientific studies on the association between social media use and well-being have reported mixed results with small effect sizes (Odgers and Jensen, 2020, Orben, 2020, Orben and Przybylski, 2019). These mixed findings and small associations might be the result of the large heterogeneity of adolescents’ social media use (Odgers and Jensen, 2020). That is, the effects of social media on well-being varies substantially between adolescents (Beyens et al., 2020). These results highlight the need to examine individual differences in the association between social media and well-being, using longitudinal data (Crone and Elzinga, 2015). Therefore, in this study we first examined heterogeneity in developmental trajectories of social media use across adolescence and subsequently tested whether individual differences in social media use across development were associated with baseline levels (intercept) and changes (slopes) in depression and anxiety, measures that are often used to indicate mental well-being (Kross et al., 2020, Orben, 2020).

Most studies to date have focused solely on the direct association between social media use and well-being, without taking into account the underlying neural correlates (Prinstein et al., 2020). Two decades of developmental neuroscience research has shown important changes in brain structure and function across the transition from childhood to adulthood. Decreases in brain grey matter volume, which can be seen as a proxy for brain maturation (Huttenlocher, 1990), continue until the mid-twenties (Gogtay et al., 2004, Mills et al., 2016, Tamnes et al., 2017). Specifically, regions important for social processing such as the medial prefrontal cortex (MPFC), posterior superior temporal sulcus (pSTS) and temporal parietal junction (TPJ), as well as regions involved with cognitive control, such as the lateral prefrontal cortex (LPFC), show extensive decreases in grey matter volume (i.e., cortical thinning) across adolescence (Becht et al., 2020, Mills et al., 2014a, Mills et al., 2014b).

Individual differences in cortical thinning have been related to behavioral outcomes. It has been suggested that negative environmental circumstances can accelerate brain maturation, whereas greater access to positive experiences decelerates maturation (Tooley et al., 2021). Several empirical studies have shown such accelerated brain maturation for behaviors associated with mental well-being and social media use. For example, accelerated cortical thinning of the prefrontal cortex has been associated with higher levels of depression within a non-clinical adolescent sample (Bos et al., 2018). Moreover, diminished cortical thickness of (amongst others) the ventral LPFC and TPJ across adolescence have been related to a lack of impulse control (Pehlivanova et al., 2018), a characteristic that has often been related to social media use. Indeed, Wilmer and Chein, 2016, Wilmer et al., 2019 reported that individual differences in smartphone use were associated with impulsivity as measured with a delay discounting task. Moreover, they found that stronger structural connectivity between ventral striatum and MPFC was related to more engagement with smartphones, whereas connectivity between ventral striatum and dorsal LPFC was related to less engagement (Wilmer et al., 2019). Moreover, Paulus and collogues (2019) used a group factor analyses to investigate latent variables that relate to social media activity and found that four group factor analyses explained 37% of the variance between social media and structural brain indices (cortical thickness, sulcus depth and volume). However, these findings all stem from cross-sectional studies and although these provide interesting insights in associations age-effects, actual individual development can only be captured using longitudinal designs (Crone and Elzinga, 2015, Steen et al., 2007).

Very little is known about the association between longitudinal brain development and social media use in adolescence (Crone and Konijn, 2018). Therefore, the second aim of this study was to examine whether heterogeneity in social media use and mental well-being were related to (similar) individual differences in structural brain development. We will specifically focus on two structural brain metrics: surface area and cortical thickness. Previous studies have shown that cortical thickness decreases linearly across adolescence, whereas surface area shows mostly non-linear developmental decreases (Mills et al., 2014a, Mills et al., 2014b). In addition, cortical thickness and surface area differ in the degree in which they are influenced by heritability. That is, surface area is more influenced by genetic variants than cortical thickness (Grasby et al., 2020). By including both metrics we can examine distinct aspects of structural brain development.

The current study thus aimed to examine longitudinal associations between structural brain development, social media use, and mental well-being during adolescence. Specifically, we examined whether the same neural mechanisms are associated with individual differences in longitudinal trajectories of both social media use as well as mental well-being. We made use of data from the accelerated longitudinal Self-Concept Study (van der Cruijsen et al., 2018)*,* which included 189 typically developing Dutch adolescents at three time points spanning approximately 5 years.

Our first preregistered aim (see Achterberg et al., 2021) was to examine heterogeneity in developmental trajectories of social media use across adolescence. First, we conducted a multivariate latent class growth curve analyses (LCGA (Jung and Wickrama, 2008)) on three waves to examine the number of subgroups and the shape of their developmental trajectories on self-reported time spent on social media and self-reported compulsive social media use. We expected to find at least two subgroups: one that shows a developmental increase in social media use (both time spent and compulsiveness) and one that shows a developmental increase in time spent on social media, but stable low compulsiveness. Next, we examined whether social media use subgroups differed on baseline (intercepts) and development (slopes) of mental well-being (self-reported levels of depression and anxiety) and fear of negative evaluation across the three waves. To this end, we conducted a series of latent growth curve models (LGMs). We expected that the subgroup with high social media use (both time spent and compulsiveness) across time will show a decrease in mental well-being over time (Twenge et al., 2018), whereas the group with increased time spent on social media and stable low compulsiveness will display higher and stable levels of mental well-being (Beyens et al., 2020, Odgers and Jensen, 2020, Orben, 2020).

The second preregistered aim (see Achterberg et al., 2021) of this study was to examine whether social media subgroups differed in their structural brain development. We specifically focused on four regions of interest important for social processing (MPFC, TPJ, pSTS) and a region more strongly involved in cognitive control (LPFC). Previous studies have shown a decrease of surface area and cortical thickness in specifically these regions across adolescence (Mills et al., 2014a, Mills et al., 2014b, Tamnes et al., 2017, Wierenga et al., 2014). Moreover, these regions have been related to social media use specifically (MPFC, LPFC, Wilmer et al., 2019) or social relations in general (TPJ, pSTS, (Becht et al., 2021, Blakemore, 2008). We therefore used these four regions as a starting point to unravel if – and how - individual differences in social media use were associated to individual differences in structural brain development. We expected that individuals with relatively high social media use (both time spent and compulsiveness) will show accelerated cortical thinning (stronger decrease of surface area and cortical thickness) in these regions compared to individuals with stable low social media compulsiveness use across time (Prinstein et al., 2020).

Exploratively, we conducted the same analyses with subgroups based on heterogeneity in mental well-being. These analyses were not part of our preregistration, but can provide important insight into whether there is an overlap in neural regions associated with changes in both social media use and well-being. Based on prior research we expected accelerated cortical thinning in the LPFC for adolescent who experience higher levels of depression and anxiety (Bos et al., 2018). We first conducted a univariate LGCA on three waves of mental well-being data to examine the number of subgroups and shape of their developmental trajectories. Next, we examined whether mental well-being subgroups differed on baseline and slopes of social media use and fear of negative evaluation across the three waves. Last, we examined whether similar associations between longitudinal structural brain development could be found based on heterogeneity in mental well-being (compared to heterogeneity in social media use). By doing so, we were able to examine whether cortical thinning of social and cognitive control regions in the brain might serve as an underlying mechanism driving both individual differences in social media use as well as individual differences in mental well-being.

## 2 Methods

### 2.1 Participants

This project is part of the larger Leiden Self-Concept study (see Open Science Framework: https://osf.io/8gc6x), in which 187 individuals were followed across three annual MRI assessments. At the first time point (T1) 160 adolescents were included (aged 11–21, mean age: 15.97 ± 2.97, 54% female), see Table 1 for demographics per age bin. To get a balanced number of participants in terms of age across waves, additional participants were included in the younger age range at T2, and in the older age range at T3. At T2, the sample included 167 adolescents (aged 10–22, mean age: 16.66 ± 3.20, 50% female). At T3, the sample included 175 participants (aged 11–24, mean age: 18.14 ± 3.42, 51% female). The vast majority of the sample (N = 111, 59%) had structural brain data at all three waves (two waves: 21%; one wave: 19%; only behavior data: 1%). Participants and parents of minors provided written informed consent. This study was approved by the Medical Ethics Committee of Leiden University Medical Center. Exclusion criteria before participation were MRI-contraindications, left-handedness and a current or previous diagnosis of a neurological or psychiatric disorder. Scans were inspected by a radiologist and no clinically relevant findings were found.

**Table 1:** Estimated intercept and slope growth parameters of the final 2-class solution for social media use. p-values indicate whether the intercept and slopes were significantly different from zero (two-tailed t-tests).

	Low Social Media Class (N = 137)			High Social Media Class (N = 52)		
	Mean	S.E	p-value	Mean	S.E.	p-value
**Social Media Time**						
intercept	**14.63**	**1.78**	**< 0.001**	**45.85**	**11.7**	**< 0.001**
linear slope	-1.58	5.25	.763	-4.17	14.42	.773
quadratic slope	1.77	2.72	.515	1.00	6.7	.882
**Social Media Compulsiveness**						
intercept	**0.201**	**0.059**	**.001**	**1.336**	**0.353**	**< 0.001**
linear slope	0.045	0.260	.862	-0.391	0.884	.659
quadratic slope	0.019	0.109	.864	0.111	0.338	.744

### 2.2 Self-reported behavioral measures

For descriptive purposes, we described age and sex effects for the self-reported behavioral measures for each of the time points separately in Table 2.

**Table 2:** Estimated intercept and slope growth parameters for mental well-being (anxiety and depression measured by RCADS) and fear of negative evaluation for each social media class.

	Low Social Media Class (N = 137)		High Social Media Class (N = 52)			
	Mean	S.E.	Mean	S.E.	Chi-square	p-value
**Mental Well-being (Anxiety and Depression)**						
intercept	0.41	0.03	0.43	0.04	0.08	0.772
linear slope	0.10	0.00	0.10	0.01	0.88	0.349
**Fear of Negative Evaluation**						
intercept	0.15	0.09	0.37	0.15	1.54	0.215
linear slope	0.61	0.03	0.55	0.05	0.75	0.388

#### 2.2.1 Time spent on social media

Time spent on social media was measured by self-report (see also (Peters et al., 2021). At each of the three time points, we asked participants two questions: 1) *“In the last two weeks, how many days have you used social media (*e.g.*, Facebook, Twitter, Instagram)?”* and 2) *“How many hours a day do you typically spent on social media (*e.g.*, Facebook, Twitter, Instagram)?”* Based on these two questions we calculated the number of hours spent on social media in last two weeks (days in the last two weeks * number of hours a day) for each of the three time points. We excluded one participant at the second wave, who reported spending 336 h on social media in the last two weeks (implicating that this participant spent 24/7 online, which we deemed unrealistic). The intraclass correlation coefficient (ICC) demonstrated moderate stability of time spent on social media across waves (ICC = 0.74).

#### 2.2.2 Compulsive social media use

Compulsive social media use was measured by self-report of the Compulsive Internet Use Scale (CIUS) (Meerkerk et al., 2009) at three time-points. In contrast to the questions regarding time spent on social media, the questions concerning compulsive social media use were specifically aimed at the use of Facebook. The CIUS consists of 14 questions (e.g., “*How often do you continue to use Facebook while you intended to quit?”* and *“How often do you think you should spend less time on Facebook?”*) which could be rated on a 5-point Likert scale ranging from 1 (never) to 5 (very often). The internal consistency of the CIUS has shown to be high (*α* = 0.90, (Meerkerk et al., 2009)). Within our sample, Cronbach’s alpha based on the 14 items was excellent for all three waves (T1: *α* = 0.93, T2: *α* = 0.92; T3: *α* = 0.94). Stability of compulsive social media use within individuals over time was moderate (ICC=0.67). We computed a mean score for compulsive social media use for each individual at each of the three time-points. Higher scores indicate more compulsive social media use.

#### 2.2.3 Mental well-being

Mental well-being was measured at all three time-points by self-report of the Dutch version of the Revised Child Anxiety and Depression Scale (RCADS (Kösters et al., 2015; Mathyssek et al., 2013)). The RCADS (Chorpita et al., 2000) indicates how often a child suffers from certain symptoms of anxiety or depressive feelings. The internal consistency of the Dutch RCADS has been shown to be good (*α*’s = 0.70–0.96) in 8–13-year-olds (Kösters et al., 2015). The RCADS consists 47 items (e.g., *“I feel sad or empty”* and *“I spend the night in bed worrying”*) which could be rated on a 4-point Likert scale (never, sometimes, often, always). Within our sample, Cronbach’s alpha based on the 47 items was excellent for all three waves (T1: *α* = 0.93, T2: *α* = 0.94; T3: *α* = 0.95). Stability of mental well-being within individuals over time was high (ICC = 0.85). We computed a mean score for mental well-being based on all the items for each individual at each of the three time-points. Higher scores indicate lower mental well-being.

#### 2.2.4 Fear of negative evaluation

Fear of social evaluation was measured using the brief version of the Fear of Negative Evaluation questionnaire (Leary, 1983). The Fear of Negative Evaluation (FNE) questionnaire measures discomfort and distress in interpersonal interactions (Watson & Friend, 1969). The brief FNE consists of 12 items (e.g., *“I rarely worry about what kind of impression I am making on someone”* and *“I am afraid others will not approve of me”*) which could be rated on a 5-point Likert scale (never, sometimes, often, always. Within our sample, Cronbach’s alpha based on the 12 items was excellent for all three waves (T1: α = 0.96, T2: α = 0.97; T3: α = 0.97). Stability within individuals over time was high (ICC=0.87). We computed a mean score based on the 12 items for each individual at each of the three time-points. Higher scores indicate more fear of negative evaluation.

### 2.3 Structural brain measures

#### 2.3.1 Data acquisition

MRI scans were acquired with a standard whole-head coil on a Philips Achieva 3.0 Tesla MRI system at Leiden University Medical Centre (LUMC). To prevent head motion, foam inserts surrounded the participant’s head. Participants could watch a movie during the scan which was projected on a screen that was visible through a mirror on the head coil. One high-resolution 3D T1-weighted scan was acquired for each participant (Field of view (FOV, in mm) = 224 (ap) x 177 (rl) x 168 (fh); TR = 9.8 ms; TE = 4.6 ms; flip angle= 8°; 140 slices; voxel size 0.875 × 0.875 × 0.875 mm). Scan time for the anatomical scan was 296 s

#### 2.3.2 Data processing

Similarly, to what has been previously reported by (Becht et al., 2020) on the same sample, cortical reconstruction was performed with the longitudinal stream (Reuter et al., 2012) in FreeSurfer 6.0.0, a program for cortical surface reconstruction and volumetric segmentation (http://surfer.nmr.mgh.harvard.edu/). The procedure and technical details are described elsewhere (Fischl et al., 1999, Fischl et al., 1999, Reuter et al., 2012). To extract reliable volume estimates, an unbiased within-subject template space and image is created using robust inverse consistent registration (Reuter et al., 2012). Several processing steps, such as skull stripping, Talairach transformation, atlas registration as well as spherical surface maps and parcellations are then initialized with common information from the within-subject template, significantly increasing reliability and statistical power (Reuter et al., 2012). Parcellation of the cortex into gyral regions was based on the Desikan-Killiany atlas (Desikan et al., 2006).

#### 2.3.3 Quality control

Post-processing of the scan quality was conducted using a hybrid manual-automatic quality assessment tool, Qoala-T (Klapwijk et al., 2019). All T1-weighted scans of the first wave (T1) were manually checked, using the protocol described in (Klapwijk et al., 2019). Next, the Qoala-T algorithm was run on all scans from T1, T2, and T3. Results of Qoala-T advised a manual quality check for nine participants at T2 and for five participants at T3. In addition, to confirm the accuracy of the model prediction, a random subset (20%) of the scores > 70 at T2 and T3 were selected for additional manual quality checks. Data of three participants were excluded at T1, two at T2 and one at T3. These data were replaced with missing values.

#### 2.3.4 Regions of interest

We investigated four regions of interest (ROIs): the medial prefrontal cortex (MPFC), temporal parietal junction (TPJ) posterior superior temporal sulcus (pSTS), and the lateral prefrontal cortex (LPFC). The MPFC, TPJ and pSTS regions are based on the social brain regions as defined in (Mills et al., 2014a, Mills et al., 2014b). Similar to previous work (Becht et al., 2020) the LPFC ROI will be defined using the Desikan-Killiany cortical parcellation atlas by combining the following subdivisions: rostral middle frontal, caudal middle frontal, caudal anterior cingulate, and superior frontal. For each ROI, we extracted measurements of surface area (SA, in mm^2^) and cortical thickness (CT, in mm). To limit the number of statistical tests, we computed bilateral estimates by combining structural measures for each hemisphere. For surface area we will use an average of the left and right hemisphere. For cortical thickness we will take the size of each region into account (also see van der Meulen et al., 2018) by using the following formula:

$$\frac{\left((left\;hemisphere\;CT*SA)+(right\;hemisphere\;CT*SA)\right)}{\left(left\;hemisphere\;SA+right\;hemisphere\;SA\right)}$$

#### 2.3.5 Univariate latent growth curve models

We conducted eight univariate latent growth curve models (Duncan and Duncan, 2009), one for each of the four ROIs multiplied by the two structural outcomes (surface area and cortical thickness). Note that previous work of Becht et al. (2020) already reported on latent growth curve models of surface area and cortical thickness for the MPFC and LPFC on the same sample.

We tested for linear and non-linear (quadratic) growth models and determined the best fitting model based on the Akaike information criteria (AIC) (Akaike, 1974); the Bayesian Information Criterion (BIC) (Schwarz, 1978), and the sample size adjusted BIC (ssaBIC) (Sclove, 1987), with lower values indicating a better model fit. As a result of the accelerated longitudinal design of the Self-concept study (van der Cruijsen et al., 2018, Becht et al., 2020), the age range is relatively wide at each time point (e.g., at T1 the age ranges from 11 to 21 years). Therefore, we used the TSCORES option in Mplus to account for individually varying times of observations (see (Mehta and West, 2000) for an in-depth discussion), similar to earlier work on the same longitudinal study (Becht et al., 2020). In all latent growth curve models, we controlled the intercepts and slopes of social media use for sex differences. In case the model fit of the final model (linear vs quadratic) did not significantly improve (based on lower AIC, BIC, ssaBIC) with the inclusion of sex as a covariate, we dropped sex as a covariate from the final growth curve models. Table 3 provides an overview of the best fitting (final) latent growth curve model for each of the four ROIs, both for surface area as well as cortical thickness. The best fitting model for surface area for all ROIs was a quadratic growth trajectory including sex as covariate. For cortical thickness, the best fitting model for LPFC, MPFC and pSTS was a quadratic growth trajectory. For TPJ cortical thickness, the quadratic model did not converge and therefore we continued our subsequent analyses with the linear trajectory as the best fitting model. For cortical thickness the inclusion of sex did not improve the model in any of the ROIs and was therefore not included in the final models. For each individual we saved the individual intercept and slopes of the final latent growth curve model. These intercepts and slopes were then used to test intercept and slope differences on brain development across subgroups, using Wald Chi-Squared tests.

**Table 3:** Estimated intercept and slope growth parameters for structural brain development for each social media class. The cut-off for significant p-values after FDR correction was *p* < .002.

	Low Social Media Class (N = 137)		High Social Media Class (N = 52)			
	Mean	S.E	Mean	S.E.	Chi-square	p-value
***Cortical Thickness***						
**Lateral Prefrontal Cortex**						
intercept	3.846	0.021	3.947	0.030	7.099	**0.008**
linear slope	-0.826	0.022	-0.914	0.033	4.438	**0.035**
quadratic slope	0.175	0.005	0.193	0.009	3.110	0.078
**Medial Prefrontal Cortex**						
intercept	4.053	0.033	4.186	0.038	6.454	**0.011**
linear slope	-0.881	0.043	-1.007	0.059	2.776	0.096
quadratic slope	0.170	0.011	0.199	0.015	2.291	0.130
**Temporal Parietal Junction**						
intercept	3.213	0.014	3.218	0.020	0.030	0.863
linear slope	-0.208	0.005	-0.221	0.006	2.368	0.124
quadratic slope	–	–	–	–	–	–
**Posterior Superior Temporal Sulcus**						
intercept	3.963	0.032	4.080	0.050	3.620	0.057
linear slope	-1.078	0.044	-1.184	0.071	1.487	0.223
quadratic slope	0.250	0.012	0.268	0.019	0.626	0.429
***Surface Area***						
**Lateral Prefrontal Cortex**						
intercept	18227.00	23.30	18077.00	37.30	0.11	0.74
linear slope	-761.00	7.80	-815.00	11.60	0.14	0.71
quadratic slope	27.00	1.20	20.00	1.60	0.12	0.74
**Medial Prefrontal Cortex**						
intercept	584.000	21.200	519.300	27.800	3.161	0.075
linear slope	42.400	26.100	104.300	36.100	1.784	0.182
quadratic slope	-17.100	7.200	-34.000	10.200	1.707	0.191
**Temporal Parietal Junction**						
intercept	2015.200	23.200	2030.800	34.200	0.132	0.716
linear slope	-238.400	14.300	-294.100	19.700	4.846	**0.028**
quadratic slope	39.600	4.400	55.300	6.300	3.880	**0.049**
**Posterior Superior Temporal Sulcus**						
intercept	1952.6	26	1885.6	36.7	2.053	0.152
linear slope	-376.1	10.4	-360	15	0.724	0.395
quadratic slope	80.5	2.2	76.4	3.1	1.134	0.287

Note: no p-values were significant after FDR correction.

### 2.4 Statistical analyses

Little’s missing completely at random (MCAR) test showed that missing data patterns of questionnaire measures and brain variables were MCAR (χ^2^(16) = 25.52, *p* = .061). As preregistered, we included all participants with and without missing values in our analyses and handled missing data using full information maximum likelihood (FIML) in Mplus (Muthén and Muthén, 1998). We controlled for multiple testing by using a false discovery rate (FDR) correction, using the Benjamini-Hochberg procedure (Benjamini and Hochberg, 1995), based on p-values per output table for Table 3, Table 5, Table 6. The cutoff for significant FDR corrected p-values are depicted in the table headings.

#### 2.4.1 Preregistered analyses (https://doi.org/10.17605/OSF.IO/DGMBX)

The first aim of this study was to examine heterogeneity in developmental trajectories of social media use across adolescence. To this end, we conducted a multivariate latent class growth curve analyses (LCGA (Jung and Wickrama, 2008)) in Mplus (Muthén and Muthén, 1998) on three waves to examine the number of subgroups and shape of their developmental trajectories of time spent on social media and the level of compulsive social media use. We could not use the TSCORES option to account for the age heterogeneity at each wave, as this was too complex for the data (i.e., too many parameter estimates relative to the number of observations, resulting in non-convergence of the LCGA). Alternatively, as preregistered, we controlled the intercept and slopes for age (see also Becht et al., 2020)). To empirically determine the number of latent classes that best fit the data, used the AIC (Akaike, 1974); BIC (Schwarz, 1978), and the ssaBIC (Sclove, 1987), with lower values indicating a better model fit. Moreover, as preregistered, every subgroup needed to cover at least 15% of the sample (N = ± 25) for meaningful interpretation and subsequent analyses. The distribution of sex across the different subgroups was examined using chi square tests.

Secondly, as preregistered, we examined whether social media use subgroups differed on baseline and slopes of mental well-being, as measured by levels of depression and anxiety across the three waves. As additional, non-preregistered behavioral measure we examined whether social media use subgroups differed on baseline and slopes of fear of negative evaluation.

Third, we tested for differences in the intercepts and slopes of the structural brain regions across the different social media subgroups. To this end, we first saved the individual intercept and slopes of the final latent growth curve models (see section 4.3.5.). Next, we tested intercept and slope differences on brain development across the identified subgroups in LCGA. To this end, we used the three step BCH approach (Asparouhov and Muthén, 2014).

#### 2.4.2 Exploratory analyses

Exploratively, we conducted the same analyses with subgroups based on heterogeneity in mental well-being. These analyses were not part of our preregistration, and no specific hypotheses were stated beforehand. We first conducted a univariate LGCA on three waves of mental well-being data to examine the number of subgroups and shape of their developmental trajectories.

Secondly, we examined whether mental well-being subgroups differed on baseline and slopes of social media use (both time spent and compulsiveness), and fear of negative evaluation across the three waves.

Third, we tested for differences in the intercepts and slopes of the structural brain regions across the different mental well-being subgroups. That is, we examined whether the same associations between heterogeneity in social media use and structural brain development could be found based on heterogeneity in mental well-being. By doing so, we were able to test whether cortical thinning of social and cognitive control regions in the brain might serve as an underlying mechanism driving both individual differences in social media use as well as individual differences in mental well-being.

### 2.5 Deviations from preregistration

#### 2.5.1 Outliers

We preregistered to not delete outliers on the brain and behavior variables in our analyses (except for the MRI scans that were of insufficient quality, see section 4.3.3.). However, we excluded data of time spent on social media for one participant at T2, as this data entrance was highly improbable (see also section 4.2.1.).

#### 2.5.2 External validity check

We preregistered to examine whether the social media subgroups differed on mental well-being (measured by levels of depression and anxiety across the three waves) as external validity check for the subgroups. In addition to this, and thus not preregistered, we also examined whether the subgroups differed on fear of negative evaluation. We chose this measure as it is more closely related to social media use and therefore might be more sensitive for class differences.

#### 2.5.3 Cuneus

We preregistered that the accelerated cortical thinning (stronger decrease in surface area and cortical thickness) for individuals with increased social media use would be specific for social brain regions (MPFC, TPJ, pSTS) and cognitive control regions (LPFC). We preregistered to also examine group differences for structural development of the cuneus (a lower-level processing visual region) as a comparison and to test for the specificity of our results. However, we deviated from the preregistration by not including the cuneus as this control region was not of primary interest for the goals of this study.

## 3 Results

### 3.1 Preregistered analyses

#### 3.1.1 Social media use classes

The first aim was to examine heterogeneity in developmental trajectories of social media use (time spent on social media and the level of compulsive social media use) across adolescence. We conducted a multivariate latent class growth curve analyses (LCGA) on three waves to examine the number of subgroups and shape of their developmental trajectories.

**Fig. 1:**
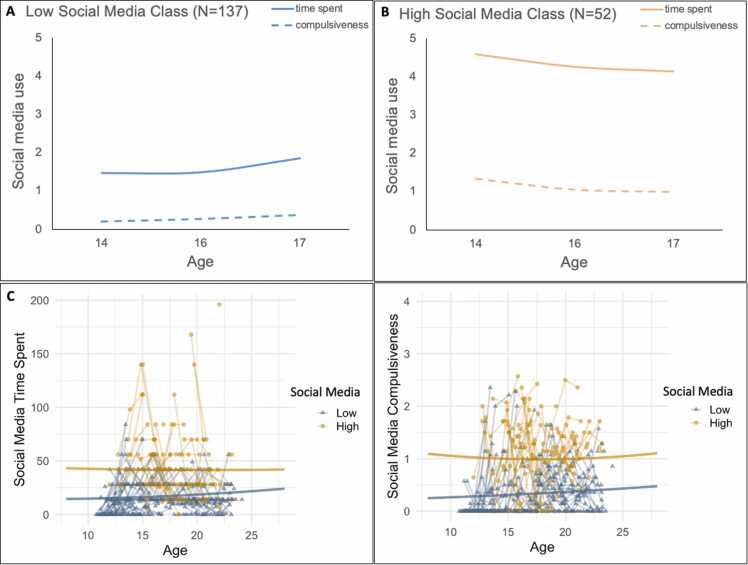
Social media use classes. The top line shows time spent on social media (hours in last two weeks*) and compulsive use of social media (measured with CIUS) for (a) the low social media class and (b) the high social media class, scaled at the average age for each of the three waves. The bottom line shows individual data for both low and high social media users for (c) time spent on social media and (d) compulsive social media use. * Note that time spent was divided by a factor of 10 in the top plots for scaling purposes.

Consistent with our pre-registered hypothesis, the LCGA revealed that a two-class solution provided the best fit to the data. That is, all three model fit evaluation criteria were lower for the two-class solution (AIC: 3825.48, BIC: 3916.25, and ssaBIC: 3827.56) compared to the one-class solution (AIC: 4063.81, BIC: 4128.65, and ssaBIC: 4065.29). Entropy (0.88) of the two-class solution was high. A three-class solution resulted in too small subsamples (i.e., one class included only 11 participants (i.e., 6% of the total sample and thus less than the pre-registered <15% of the sample). Therefore, we continued our analyses using the two-class solution (Fig. 1).

The first social media class (N = 137, 72%) was characterized by i) relatively low and stable levels of time spent on social media across development, and ii) relatively low and stable compulsive social media use (Fig. 1a). The second social media class (N = 52, 28%) was characterized by i) relatively high and stable time spent on social media, and ii) relatively high and stable compulsive social media use (Fig. 1**b**). For comparison and overview purposes, we will refer to the first group as Low Social Media Use and the second group as High Social Media Use. Note that these groups were not compulsively high or low on social media use. The estimated intercept and slope growth parameters of the final 2-class solution can be found in Table 4.

**Table 4:** Estimated intercept and slope growth parameters of the final 2-class solution for mental well-being. p-values indicate whether the intercept and slopes were significantly different from zero (two-tailed t-tests).

	High Mental Well-being (N = 155)			Low Mental Well-being (N = 34)		
	Mean	S.E	p-value	Mean	S.E.	p-value
**Mental Well-being (Anxiety and Depression)**						
intercept	**0.45**	**0.05**	**< 0.001**	0.95 *	**0.09**	**< 0.001**
linear slope	-0.04	0.05	0.386	0.10	0.16	0.527
quadratic slope	0.04	0.02	0.051	-0.01	0.07	0.927

Although the two-class solution is consistent with our pre-registered hypothesis, the trajectories of the subgroups did not exactly match our expectations. That is, we expected that the time spent on social media would increase in both groups, but our analyses show stable time spent on social media across adolescence (i.e., no significant slope effects, see Table 4). Moreover, we expected increased compulsiveness in the high social media subgroup and stable compulsiveness in the low social media group, however our results indicated stable social media compulsiveness across adolescence (i.e., no significant slope effects in both groups, see Table 4). In the remaining manuscript we rephrased our hypothesis such that the hypothesized subgroups correspond to the subgroups revealed by the LCGA.

Individual data for both low and high social media users are visualized for time spent on social media (Fig. 1c) and social media compulsiveness (Fig. 1d). There were significant sex differences in the class distribution (*χ*^2^ =7.83, *p* = .006). Whereas the low social media class had equal sex distribution (55% male, 45% female), the high social media class included more females (68%) than males (32%). Moreover, the high social media subgroup was significantly older at the first measurement (*M*=16.71, *SD*=2.32) than the low social media subgroup (*M* = 15.64, *SD* = 3.16, *t*(158) = −2.12, *p* = .036)). There were no significant differences in IQ between high and low social media users (*t*(158) = 1.51, *p* = .132).

#### 3.1.2 Behavioral differences between social media classes

**Fig. 2:**
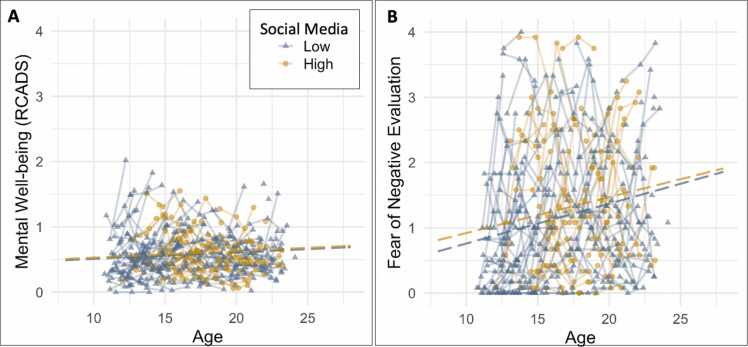
There were no significant differences between the high and low social media use class on (a) mental well-being (measured with anxiety and depression) and (b) Fear of negative evaluation. Dashed lines represent no group differences.

As external validity check of the classes we examined whether the social media subgroups differed on mental well-being, as measured by levels of depression and anxiety across the three waves. Contrary to our hypothesis, we found no significant intercept or slope differences between high and low social media use classes for anxiety and depression. (Fig. 2a, Table 5). As an additional (non-preregistered) validity check, we also examined whether the social media classes differed on fear of negative evaluation. We chose this measure as it is more closely related to social media use and therefore might be more sensitive for class differences. However, although we report a significant linear increase with age for fear of negative evaluation, we found no significant intercept or slope differences between high and low social media use (Fig. 2b, Table 5).

**Table 5:** Estimated intercept and slope growth parameters for time spent on social media, social media compulsiveness, and fear of negative evaluation for each mental well-being class. The cut-off for significant p-values after FDR correction was *p* < .016.

	High Mental Well-being (N = 155)		Low Mental Well-being (N = 34)			
	Mean	S.E	Mean	S.E.	Chi-square	p-value
**Social Media Time**						
intercept	0.70	0.28	-0.96	1.06	2.14	0.144
linear slope	13.28	0.83	18.83	0.31	2.70	0.100
**Social Media Compulsiveness**						
intercept	0.52	0.03	0.55	0.08	0.11	0.740
linear slope	0.01	0.01	-0.01	0.02	0.50	0.481
**Fear of Negative Evaluation**						
intercept	0.85	0.06	2.07	0.16	45.89	**< 0.001**^a^
linear slope	0.10	0.02	0.15	0.04	1.62	0.203

a p-value significant after FDR correction.

#### 3.1.3 Structural brain differences between social media classes

Our second aim was to test whether the two social media classes differed in the structural brain development of LPFC, MPFC, TPJ and pSTS. We hypothesized that individuals with high social media use would show stronger decrease of surface area and cortical thickness in these regions compared to individuals with stable low social media use across time. Similar to our analyses on behavioral differences between the groups, we extracted the intercepts and slopes for each individual from the latent growth curve models and tested whether there were significant group differences. We examined these group differences on cortical thickness and surface area, resulting in eight group comparisons (Table 6, Fig. S1). None of the effects survived FDR correction for multiple testing (*p* < .002).

**Table 6:** Estimated intercept and slope growth parameters for structural brain development for each mental well-being class. The cut-off for significant p-values after FDR correction was *p* < .013.

	High Mental Well-being (N = 155)		Low Mental Well-being (N = 34)			
	Mean	S.E	Mean	S.E.	Chi-square	p-value
***Cortical Thickness***						
**Lateral Prefrontal Cortex**						
intercept	3.871	0.019	3.897	0.051	0.216	0.642
linear slope	-0.844	0.020	-0.884	0.049	0.520	0.471
quadratic slope	0.178	0.005	0.188	0.012	0.534	0.465
**Medial Prefrontal Cortex**						
intercept	4.079	0.029	4.144	0.064	0.793	0.373
linear slope	-0.903	0.040	-0.977	0.076	0.691	0.406
quadratic slope	0.175	0.010	0.192	0.019	0.593	0.441
**Temporal Parietal Junction**						
intercept	3.215	0.013	3.212	0.026	0.012	0.913
linear slope	-0.210	0.004	-0.220	0.008	1.169	0.280
quadratic slope	–	–	–	–	–	–
**Posterior Superior Temporal Sulcus**						
intercept	3.977	0.031	4.083	0.060	2.269	0.132
linear slope	-1.084	0.043	-1.210	0.076	1.922	0.166
quadratic slope	0.250	0.012	0.277	0.020	1.265	0.261
***Surface Area***						
**Lateral Prefrontal Cortex**						
intercept	18,354.00	221.00	17,486.00	441.00	2.840	0.092
linear slope	-842.00	71.00	-512.00	159.00	3.313	0.069
quadratic slope	38.00	11.00	-28.00	24.00	5.997	**0.014**
**Medial Prefrontal Cortex**						
intercept	597.700	18.300	432.000	42.700	11.722	**0.001**^a^
linear slope	26.000	23.000	201.100	53.000	8.453	**0.004**^a^
quadratic slope	-12.600	6.400	-60.400	14.600	8.302	**0.004**^a^
**Temporal Parietal Junction**						
intercept	2031.400	20.700	1972.600	52.200	1.011	0.315
linear slope	-259.700	12.900	-234.300	30.600	0.538	0.463
quadratic slope	45.600	4.000	38.700	9.000	0.455	0.500
**Posterior Superior Temporal Sulcus**						
intercept	1957.600	23.900	1832.600	46.900	5.169	**0.023**
linear slope	-386.200	9.200	-311.300	21.400	9.565	**0.002**^a^
quadratic slope	82.700	1.900	65.800	4.500	11.230	**0.001**^a^

a p-value significant after FDR correction.

##### 3.1.3.1 Cortical thickness

**Fig. 3:**
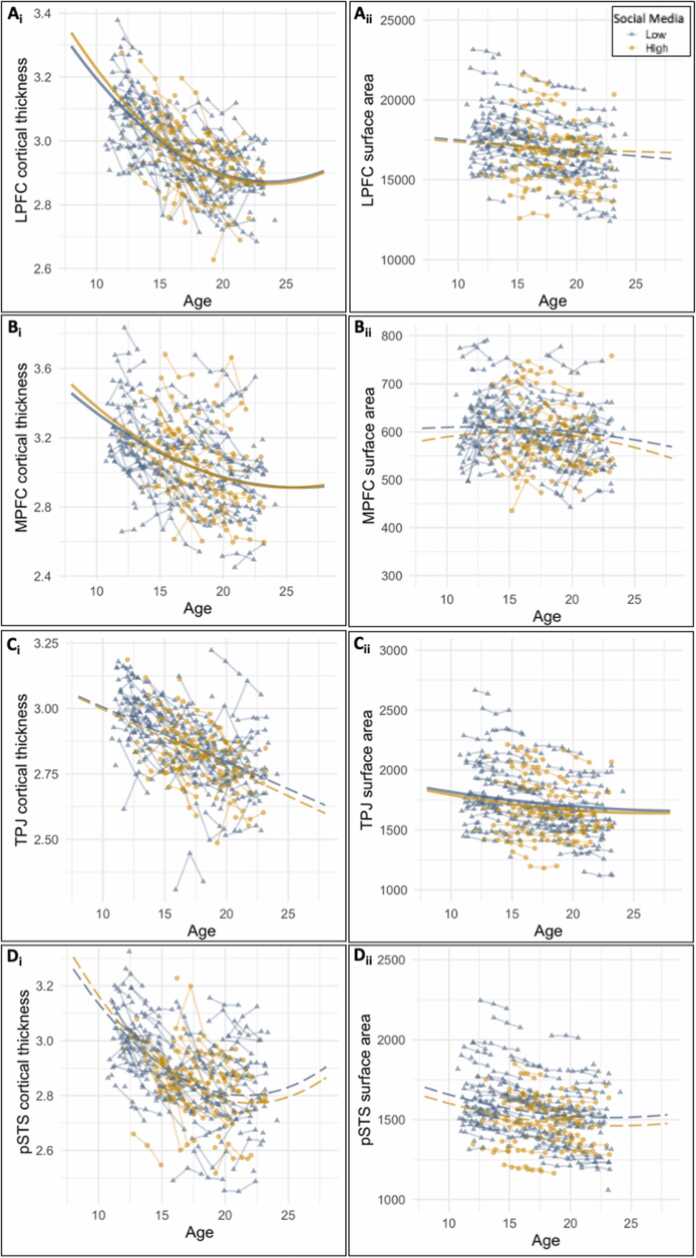
Structural brain development differences between low and high social media users for (a) lateral prefrontal cortex (LPFC), (b) medial prefrontal cortex (MPFC), (c) temporal parietal junction (TPJ), and (d) posterior superior temporal sulcus (pSTS). The left panel (i) shows cortical thickness and the right panel (ii) shows surface area. Solid lines represent significant group differences, dashed lines represent no group differences.

We found significant group differences in baseline levels of LPFC cortical thickness (intercept: *χ*^2^ =7.10, *p* = .008), where high social media users showed higher cortical thickness than low social media users (Fig. 3**a**). Moreover, adolescents with high social media use showed a stronger linear decrease across development than adolescents with low social media use (*χ*^2^ =4.438, *p* = .035). Thus, in line with our hypothesis, we found accelerated cortical thinning for individuals with high social media use in the LPFC.

We found similar effects for the MPFC: adolescents with high social media use showed higher baseline cortical thickness than adolescents with low social media use (intercept: *χ*^2^ = 6.45, *p* = .011), see Fig. 3b. The pattern of accelerated linear cortical thinning for high social media use was not significant for the MPFC (*χ*^2^ =2.78, *p* = .096). For TPJ and pSTS, we did not find significant group differences in cortical thickness (Table 6).

##### 3.1.3.2 Surface area

We found no significant group differences for LPFC, MPFC and pSTS. For the TPJ surface area, there was no significant differences in intercept (*p* = .716), but in line with our hypothesis, we did find accelerated cortical thinning. That is, adolescents with high social media use showed a stronger linear decease and a stronger quadratic increase than individuals with low social media use (linear: *χ*^2^ =4.85, *p* = .028; quadratic: *χ*^2^ =3.88, *p* = .049), (Table 6).

### 3.2 Exploratory analyses

Exploratively, we conducted the same analyses with subgroups based on heterogeneity in mental well-being. By doing so, we were able to test whether cortical thinning of social and cognitive control regions in the brain might serve as similar neural correlates associated with both individual differences in social media use as well as individual differences in mental well-being.

#### 3.2.1 Mental well-being classes

**Fig. 4:**
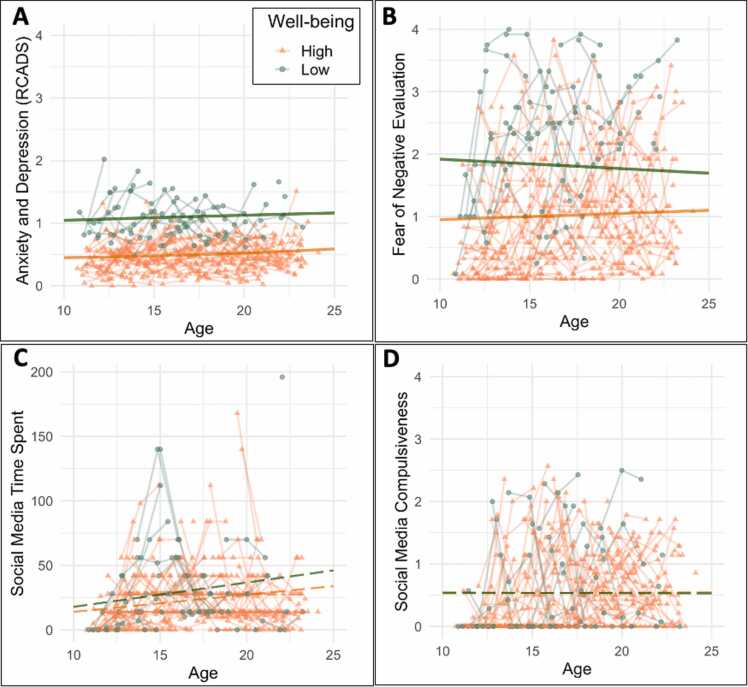
Developmental trajectories for mental well-being classes for a) anxiety and depression (input of classification), b) fear of negative evaluation, c) time spent on social media (hours in last two weeks), and d) social media compulsiveness. Solid lines represent significant group differences. Note that the developmental trajectories for high and low mental well-being completely overlap in Fig. 4d, thereby omitting the estimate of high mental well-being in the figure.

We first performed a univariate LCGA on three waves of RCADS data to examine the number of subgroups and shape of their developmental trajectories. The LCGA revealed that a two-class solution, without controlling the intercept and slopes for age, provided the best fit to the data. That is, all three evaluation criteria were lower for the two-class solution (AIC: 193.25, BIC: 225.67, and ssBIC: 193.97) compared to the one-class solution (AIC: 338.46, BIC: 357.91, and ssBIC: 338.91). Entropy (0.85) of the two-class solution was high. A three-class model solution did not converge because the third class included an empty class with 0 cases. Therefore, we continued our analyses with the final two-class solution (Fig. 4a).

The first mental well-being class (N = 155, 82%) is characterized by stable and low levels of anxiety and depression (Fig. 4a). The second mental well-being class (N = 34, 18%) is characterized by higher and stable levels of anxiety and depression (Fig. 4a). For comparison and overview purposes, we will refer to the first group as High Mental Well-being and the second group a*s* Low Mental Well-being. The estimated intercept and slope growth parameters of the final 2-class solution can be found in Table 7. There were significant sex differences in the class distribution (*χ*^2^ =8.57, *p* = .003). Whereas the high mental well-being class had equal sex distribution (54% male, 46% female), the low mental well-being class included more females (74%) than males (26%). There were no significant differences in age at the first measurement or IQ between low and high mental well-being.

**Table 7:** Demographic characteristics per age bin (wave 1). Note that age was used continuously in all analyses and the bins are only for descriptive purposes.

Age group	N	% male	mean IQ	% high educated parent (s)^a^
11–13 y.o.	50	48%	110 (10.95))	84%
14–17 y.o.	63	44%	109 (11.59)	83%
18–21 y.o.	47	47%	111 (10.44)	83%

a One or both parents completed higher vocational education or academic education

#### 3.2.2 Behavioral differences between mental well-being classes

On a behavioral level, we first examined whether the mental well-being subgroups differed on social media use, both the amount of time spent on social media as social media compulsiveness. As depicted in Table 5, we found no significant differences in social media use for high and low mental well-being classes (see Fig. 4c-d). We also examined whether there would be group differences in the fear of negative evaluation, as this measure might be more directly related to mental well-being. Indeed, we found significant group differences, such that individuals with low mental well-being had significantly higher intercepts on fear of negative evaluation than individuals with high mental well-being (*χ*^2^ =45.89, *p* < .001), see Table 8 and Fig. 4b.

**Table 8:** Age and sex effects of the behavioral measures for each of the timepoints separately.

		Age effects		Sex effects					
		r	p-value	Boys (Mean/SD)		Girls (Mean/SD)		t-value	p-value
Time spent social media	T1	**0.18**	**.021**	20.41	26.43	27.57	-25.38	-1.75	.083
	T2	0.12	.122	**15.44**	**15.71**	**30.09**	**-24.52**	**-4.55**	**< 0.001**
	T3	-0.02	.765	**18.62**	**25.72**	**30.78**	**-23.58**	**-3.25**	**.001**
Compulsive social media use	T1	**0.28**	**< 0.001**	**0.40**	**0.57**	**0.68**	**0.67**	**-2.87**	**.005**
	T2	**0.38**	**< 0.001**	**0.37**	**0.53**	**0.62**	**0.66**	**-2.66**	**.009**
	T3	0.11	.161	**0.42**	**0.61**	**0.65**	**0.70**	**-2.34**	**.020**
Anxiety and Depression (RCADS)	T1	0.05	.563	**0.46**	**0.27**	**0.60**	**0.32**	**-2.94**	**.004**
	T2	-0.04	.593	**0.46**	**0.26**	**0.67**	**0.35**	**-4.34**	**< 0.001**
	T3	-0.10	.208	**0.58**	**0.39**	**0.70**	**0.37**	**-2.15**	**.033**
Fear of Negative evaluation	T1	0.11	.180	**0.79**	**0.80**	**1.40**	**1.07**	**-4.08**	**< 0.001**
	T2	**0.19**	**.017**	**0.83**	**0.85**	**1.56**	**1.14**	**-4.65**	**< 0.001**
	T3	0.12	.117	**1.06**	**0.93**	**1.59**	**1.02**	**-3.56**	**< 0.001**

#### 3.2.3 Structural brain differences between mental well-being classes

**Fig. 5:**
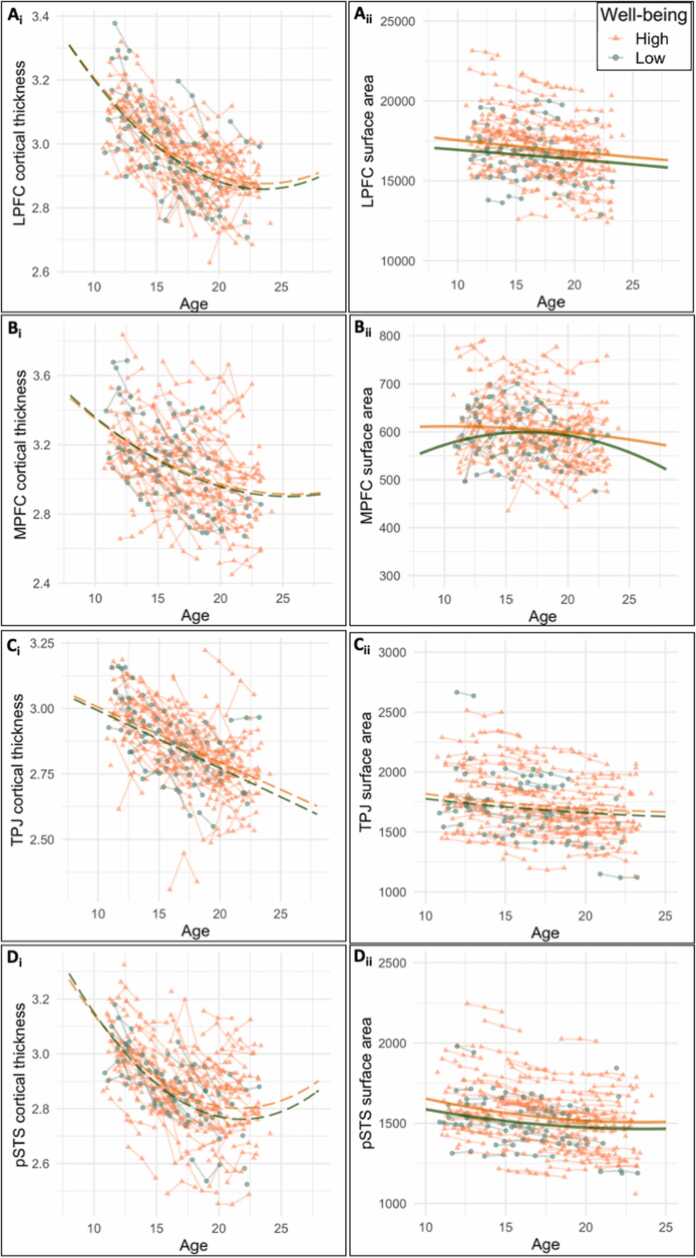
Structural brain development differences between high and low mental well-being for (a) lateral prefrontal cortex (LPFC), (b) medial prefrontal cortex (MPFC), (c) temporal parietal junction (TPJ), and (d) posterior superior temporal sulcus (pSTS). The left panel (i) shows cortical thickness and the right panel (ii) shows surface area. Solid lines represent significant group differences, dashed lines represent no group differences.

Similar to our analyses on social media classes, we tested whether the two mental well-being classes differed in the structural brain development of LPFC, MPFC, TPJ and pSTS. We examined these group differences on cortical thickness and surface area, resulting in eight group comparisons (Fig. 5). We found no significant group differences in cortical thickness in any of the four ROIs (Table 9). For surface area, there were significant group differences between low and high mental well-being in the LPFC, MPFC and pSTS (but not for TPJ, see Table 9). Only the effects with *p* < .013 survived FDR correction for multiple testing (Table 9).

**Table 9:** Fit indices of the latent growth models for surface area (SA) and cortical thickness (CT) of lateral prefrontal cortex (LPFC), medial prefrontal cortex (MPFC), temporal parietal junction (TPJ), and posterior superior temporal sulcus (pSTS). Bold formatting indicates the best fitting model.

			Lineair model			Quadratic model			model including sex as covariate		
		Best fitting model	AIC	BIC	ssaBIC	AIC	BIC	ssaBIC	AIC	BIC	ssaBIC
LPFC	SA	Quadratic incl sex	738.58	757.97	738.96	745.74	778.05	746.37	**702.68**	**744.68**	**703.50**
	CT	Quadratic	-1125.04	-1105.66	-1124.66	**-1144.56**	**-1112.25**	**-1143.92**	-1142.14	-1100.14	-1141.32
MPFC	SA	Quadratic incl sex	442.92	462.31	443.31	440.23	472.54	440.87	**378.95**	**420.95**	**379.78**
	CT	Quadratic	-601.95	**-582.56**	-601.57	**-610.40**	-578.09	**-609.76**	-607.16	-565.16	-606.34
TPJ	SA	Quadratic incl sex	840.79	860.18	841.17	834.83	867.14	835.47	**804.72**	**846.72**	**805.55**
	CT	Lineair	**-987.12**	**-967.73**	**-986.74**	a	a	a	-985.55	-959.70	-985.04
pSTS	SA	Quadratic incl sex	531.59	550.98	531.97	501.43	533.74	502.06	**454.43**	**496.43**	**455.25**
	CT	Quadratic	-978.58	-959.19	-978.20	**-1029.12**	**-996.81**	**-1028.48**	-1023.74	-981.74	-1022.91

a Model did not converge.

For LPFC surface area development, adolescents with high mental well-being showed a positive quadratic slope, whereas adolescents with low mental well-being showed a negative quadratic slope (*χ*^2^ = 5.997, *p* = .014) (Table 9, Fig. 5d). There were no significant intercept or linear slope differences between low and high mental well-being on LPFC surface area.

For MPFC surface area development, we found significant differences between low and high mental well-being on intercept, linear- and quadratic slope (Table 9). Adolescents with low mental well-being had lower baseline surface area (intercept: *χ*^2^ =11.72, *p* = .001) and a stronger linear increase across adolescence (linear slope: *χ*^2^ =8.45, *p* = .004). Moreover, adolescents with low mental well-being showed a positive quadratic slope, whereas adolescents with low mental well-being showed a negative quadratic slope (*χ*^2^ =8.30, *p* = .004), see Fig. 5e.

For pSTS surface area development, we also found significant differences between high and low mental well-being on intercept, linear- and quadratic slope (Table 9). Adolescents with low mental well-being had lower baseline surface area (intercept: *χ*^2^ =5.17, *p* = .023) than adolescents with high mental well-being, and a weaker linear decrease across adolescence (linear slope: *χ*^2^ =9.57, *p* = .002). Moreover, adolescents with low mental well-being showed a weaker quadratic slope, relative to adolescents with high mental well-being (*χ*^2^ = 11.23, *p* = .001), see Fig. 3f.

## 4 Discussion

As many as 96% of the adolescents use social media on a daily basis (Odgers and Robb, 2020), yet little is known about the impact on this intense social connectedness on adolescent development. Specifically, it is unclear how social media and ongoing brain development are associated throughout adolescence. Here, we investigated longitudinal associations between social media use, mental well-being and structural brain development across three annual assessments using a cohort-sequential longitudinal design including 189 participants with three measures across adolescence (10–25 years), as this is a time with increased emphasis on social connections. Specifically, we tested whether heterogeneity in social media use and mental well-being were related to (similar) individual differences in structural brain development.

### 4.1 Stable social media use across adolescence

Despite the increased interest in and concerns about the effects of adolescent social media use, very little research has been conducted on within-person change of social media use across development. As expected, we report evidence for heterogeneity in intercepts of time spent on social media, however unexpectedly, our results indicated that the time spent on social media within-individuals was stable across adolescence, both in the high as well as in the low social media use class. Similarly, for compulsive social media use, while we reported significant differences in intercepts of compulsiveness between the classes, our results indicated stable compulsiveness across development. One potential explanation for this stability might lay in that we specifically aimed our compulsiveness questions towards Facebook use, while over the years (i.e., 2016–2018) other platforms such as Instagram, Twitter and Snapchat increased in popularity (Ortiz-Ospina, 2019). Therefore, our measure of might have underestimated the compulsiveness of social media use. On the other hand, these results might indicate that some adolescent are more sensitive to social media use than others, and this pattern already emerges early in adolescence.

### 4.2 Structural brain development and social media use

Negative contextual factors have been suggested to accelerate brain development (Tooley et al., 2021). As compulsive social media use often is interpreted as negative contextual factor, we hypothesized that individuals with high social media use would show a stronger decrease of surface area and cortical thickness (in the LPFC, MPFC, TPJ and pSTS) compared to individuals with stable low social media use across time. We report weak structural brain differences between high and low social media users: higher social media use across time was related to higher baseline (intercept) cortical thickness in LPFC and MPFC, and individuals with high social media use showed stronger decreases in the LPFC (cortical thickness) and TPJ (surface area), compared to individuals with stable low social media use across time. None of the significant differences in brain development between high and low social media users survived FDR correction for multiple testing.

Baseline cortical thickness differences between high and low social media subgroups suggest that these differences may exist prior to adolescence. Youth that is more inclined to use social media might have different baseline cortical thickness due to, for example, an increased sensitivity to social cues in general, and these differences might increase or decrease the individual differences across development. There is some support for the latter, as a recent study using the same sample showed that better friendship quality was also related to higher baseline levels of MPFC cortical thickness (Becht et al., 2021). Better friendship quality and social media use might both be driven by individual differences in social competence and associated structural brain anatomy. Future studies should investigate whether individual differences in social competence might moderate the associations between friendship quality, social media use and structural brain development.

Alternatively, social media use early in development may influence brain development. Evidence for this hypothesis comes from accelerated cortical thinning across adolescence for high versus slow social media users. That is, we report slope differences (but not surviving multiple testing correction), where high social media users show a stronger decrease of cortical thickness in LPFC and a stronger decrease of surface area in TPJ, compared to low social media users. Several theoretical models(Snell-Rood and Snell-Rood, 2020; Tooley et al., 2021) have suggested that greater exposure to negative environmental influences such as stress could accelerate brain maturation, which might be adaptive under such circumstances. The high social media use group showed higher levels of compulsive social media use, which might be associated with increased levels of stress. However, increased levels of stress might also be a moderator driving both accelerated brain maturation as well as increased social media use, as several studies have shown that social media is often used for online help-seeking (Pretorius et al., 2019). Interestingly, the associations between social media and structural brain development were in general observed in cortical thickness development. As cortical thickness has been shown to be less influenced by genetics than surface area (Grasby et al., 2020), this might suggest that social media usage is an important environmental factor that could potentially be associated with developmental pathways.

It should be noted that the effects we report are small and, for social media subgroup analyses, do not survive correction for multiple testing. So, despite the increasing worry about the effects of social media use on developing adolescents, our longitudinal approach shows that in general, social media use is stable across adolescence. Although the results show subtle intercept and slope differences in structural brain anatomy between high and low social media users, there is little evidence for severe negative consequences of social media use on brain development. The subtle differences in longitudinal brain development trajectories might reflect individual differences in trait specific social competence as these differences seem to exist prior to adolescence. Moreover, such traits might also be related to mental well-being, which could partly explain why social media and mental well-being are sometimes (but not always) associated (Odgers and Jensen, 2020, Orben, 2020, Orben and Przybylski, 2019).

### 4.3 Social media, mental well-being and distinct brain development

We found no evidence for a direct relation between social media use and mental well-being in the current community sample. These findings are in line with systematic reviews that often report twice as many nonsignificant associations than positive associations (for example, see (Seabrook et al., 2016)). Despite finding heterogeneity in social media use, this heterogeneity was not associated with general levels of depression and anxiety, nor with specific fear towards negative evaluation. We did report significant differences in fear of negative evaluation, such that the low mental well-being class reported significantly higher (intercept) fear of negative evaluation than the high mental well-being class. This provides some evidence that mental well-being is related to social evaluation, but our data did not indicate a direct link between mental well-being and social media use in general.

In addition to prior literature that examined direct associations between social media use and mental well-being, the current study was novel in that we also investigated the relation between both processes and associated brain development. That is, we conducted the same analyses with subgroups based on heterogeneity in social media use and subgroups based on heterogeneity in mental well-being. By doing so, we were able to examine whether individual differences in structural brain maturation might serve as an underlying mechanism driving both individual differences in social media use as well as individual differences in mental well-being. In line with theoretical models and empirical studies that showed negative contextual factors can accelerate brain development (Bos et al., 2018, Snell-Rood and Snell-Rood, 2020, Tooley et al., 2021), we hypothesized accelerated cortical thinning for adolescent who experience higher levels of depression and anxiety (i.e., lower mental well-being). However - in contrast to the weak associations we found with social media use, we report no associations between mental well-being and cortical thickness. That is, for mental well-being we found solely effects on surface area development.

Individuals with low mental well-being showed significantly lower baseline surface area in the MPFC and the pSTS, surviving multiple testing correction. Moreover, the low mental well-being subgroup showed a stronger quadratic trajectory of surface area development, with a stronger increase before, and a stronger decrease after, the peak in late adolescence. We also report significant slope differences for pSTS surface area development, showing that relations between well-being and brain development were also observed in social brain regions (Blakemore, 2012). Surface area development has been shown to be sensitive to genetic variants (Grasby et al., 2020). As both anxiety and depression (which we used as measures of mental well-being) have been shown to be heritable, the associations between mental well-being and surface area might stem from genetic factors, whereas associations between structural brain development and cortical thickness might be more influenced by environmental factors. Future studies, using longitudinal twin designs and bivariate behavioral genetic modelling should elaborate on these findings (Crone et al., 2020; van der Meulen et al., 2020).

This is the first study to show that maturation of cortical brain regions is related to both social media use (weakly, not surviving FDR correction), as well as mental well-being (more strongly, surviving FDR correction). However, the associations are clearly differential: whereas social media use was related to intercept and slope differences in mostly cortical thickness, mental well-being was related to differences in surface area only. Interestingly, a recent meta-analysis showed that cortical thickness, relative to surface area, was less influenced by genetic variants(Grasby et al., 2020), suggesting that changes in cortical thickness might be more sensitive to contextual, social, environmental influences, such as social media use (Ferschmann et al., 2021). Moreover, depression and anxiety are known to be heritable, and such genetic predisposition could possibly also influence genetically driven individual differences in surface area. Besides differences in structural brain metrics, we also found associations in distinct brain regions. Although both social media use and mental well-being were associated with LPFC and MPFC development, social media use was additionally associated with the TPJ development, whereas mental well-being was associated with differences in pSTS development. Thus, although social media use and mental well-being are both associated with differences in brain development, the associations are distinct. Possibly these two processes co-exist in time but follow separate developmental trajectories.

### 4.4 Methodological considerations and future directions

This is the first study to investigate longitudinal associations between social media use, mental well-being and structural brain development across adolescence. Whereas previous studies examined social media use across a small age range using cross-sectional designs (Paulus et al., 2019) or longitudinal experience sample methods (ESM) across a narrow time span (Beyens et al., 2020) we examined the developmental trajectory of social media use using a cohort-sequential design across the whole span of adolescence (10–25 years), including three annual measures per individual. Moreover, as suggested by recent theoretical perspectives (Prinstein et al., 2020) we showed that individual differences in cortical thinning across adolescence were related to heterogeneity in both social media use and mental well-being, albeit in differential brain regions and metrics. Despite these strengths, there are several methodological considerations that should be taken into account for future research.

#### 4.4.1 Socio-economic status

We report no associations between social media use and mental well-being, whereas prior studies did report small, but statistically significant, associations between social media usage and depressive symptoms (for a systematic review, see (McCrae et al., 2017)). Possibly, the differences are associated with sample characteristics. That is, screen activity (such as social media use) has been associated with several socio-demographic variables. Parents of youth with higher screen activity were slightly younger, less well educated, less likely to be married and had lower household income (Paulus et al., 2019). Moreover, Black and Hispanic youth reported more screen time than White and Asian youth (Paulus et al., 2019). Our Dutch sample of mostly White adolescents with moderate to highly educated parents (Table 1) therefore might not include specific subsamples that show a negative association between social media use and mental well-being. In general, our sample scored relatively high on mental well-being, which is in line with reports showing that Dutch youth in general report high mental well-being (Currie et al., 2012, Inchley et al., 2020). Thus, the association between social media use and mental well-being might be moderated by socio-demographic variables and future research should aim to distinguish under which circumstances social media use and mental well-being might negatively influence each other, and in which circumstances they might reinforce each other.

#### 4.4.2 Social media measures

As usual in the social media literature, the time participants spent on social media was based on retrospective self-report. However, recent publications have pointed out that self-reported internet use does not provide a reliable reflection of actual use (Scharkow, 2016) and more objective measures of time spent on social media, such as smartphone software reports (Markowetz et al., 2014), might provide a more accurate indication of social media use. However, these measures have their own shortcomings, as these can only record screen time of one device (Kaye et al., 2020), whereas social media is often used on multiple devices. Moreover, self-reported measures can additionally provide an index of the subjective experience of social media use, for example in terms of compulsiveness. Our descriptive results displayed sex effects in social media use (Table 2), with females reporting higher compulsive Facebook use and more time spent on social media. One potential explanation for lower estimated social media use in males might originate from the way we targeted social media, which was solely aimed at profile platforms (i.e., Twitter, Facebook, Instagram). Studies have shown, however, that males are more likely to turn to online videogames (Fam, 2018, Greenberg et al., 2010). Through the chat option and multi-video player, online video gaming can also be seen as a form of social media (Peña and Hancock, 2006). Additional descriptive analyses on data that we did not include in the preregistration or the longitudinal analyses showed that in our sample females were indeed more likely to report compulsiveness on Facebook, whereas males were more likely to report compulsiveness with Online Gaming (see Supplementary materials). These results indicate that an important dimension of (predominantly) male socialization might be missing in the current study. To truly unravel the impact of complex social behavior such as social media use, future research should include multiple measures, such as objective screen time, distinctions between different activities and motivations and the subjective experience of social media (Aalbers et al., 2019, Beyens et al., 2020, Kaye et al., 2020). The current study made a start for this by defining social media use based on multivariate classification including time spent on social media as well as a measure of subjective compulsiveness.

#### 4.4.3 Mental well-being measure

Whereas most studies - including this one- on adolescent well-being have mostly focused on mental well-being, (i.e., the level of depression and anxiety as measured with questionnaires (Orben, 2020)), it is relevant for typically developing adolescents to investigate a broader scope of social well-being, including peer relations; self-concept; school (stress); parental support and resilience (Kross et al., 2020). Indeed, a recent report of Unicef showed that, despite the overall high mental well-being in Dutch youth, most adolescents experience high levels of school stress (Kleinjan et al., 2020). By examining a boarder scope of social well-being, the subtle but relevant associations between social media use and social well-being might be detected.

#### 4.4.4 Multiple testing and statistical power

Although we preregistered most of our analyses and hypotheses, we included many analyses and tests. We used a false discovery rate (FDR) correction to control for the proportion off type I errors. Most of our findings on social media subgroups did not survive the FDR correction and replication studies, preferably using larger samples that are more sensitive to pick up subtle brain-behavior associations are necessary to confirm our conclusions (Button et al., 2013). Moreover, our latent class growth analyses resulted in unequal groups in terms of sample size. Although the smaller classes (low social media use (N = 52) and low mental well-(N = 34) covered more than 15% of the total sample, which we preregistered as the minimum threshold to have meaningful interpretation of the results. However, our relatively small sample size may have been slightly underpowered to detect additional associations. Nevertheless, our results provide a starting point in generating more specific hypothesis to further unravel the underlying brain mechanisms that are associated with individual differences in both social media use and mental well-being. Future studies should aim to replicate these findings using larger samples, for example using the U.S. Adolescent Brain Cognitive Development (ABCD) study (Paulus et al., 2019*;* Volkow et al., 2018)*.*

## 5 Conclusions

The current study examined whether the same neural mechanisms are associated with individual differences in longitudinal trajectories of both social media use as well as mental well-being. Using latent class growth analyses, we report heterogeneity in social media use as well as mental well-being. Even though there was no direct association between social media use and mental well-being, heterogeneity in social media use and mental well-being were both associated with individual differences in structural brain development. Adolescents with high social media use showed higher baseline cortical thickness in LPFC and MPFC and stronger decreases in LPFC and TPJ – although these effects did not survive FDR correction for multiple testing. Mental well-being was also related to differences in structural brain development, but this was reflected in surface area rather than cortical thickness. The majority of results for well-being remained significant after FDR correction, indicating stronger associations. As surface area has been shown to be more sensitive to genetic variants than cortical thickness (Grasby et al., 2020), these results suggest that mental well-being and associated brain development might be more inclined to genetic factors, whereas changes in cortical thickness and social media use are possibly more strongly associated with environmental cues. Our results show the importance of examining individual difference in brain maturation and provide a starting point to further examine neural mechanisms that could explain which adolescents thrive by social media and which might be harmed.

## CRediT authorship contribution statement

M.A., A.B., and E.C. designed the research. R.C., and J.S. conducted the data collection. E.K. carried out the quality control of structural brain data. A.B. conducted the statistical analyses in Mplus. M.A. conducted the data visualization in R. M.A., A.B., R.C., I.G., J.S., E.K., E.A.C. were involved in data interpretation. M.A. wrote the manuscript with assistance from A.B. and E.A.C. R.C., I.G., J.P.S., E.K. provided feedback on the manuscript. All authors approved the submitted version of the manuscript.

## Declaration of Competing Interest

The authors declare that they have no known competing financial interests or personal relationships that could have appeared to influence the work reported in this paper.

## Data Availability

Data, study materials and analysis scripts are available at DataverseNL through https://doi.org/xx.
