# Supplementary material for: Longitudinal associations between social media use, mental well-being and structural brain development across adolescence
Source: Dev Cogn Neurosci. 2022 Feb 19;54:101088. doi: 10.1016/j.dcn.2022.101088 (PMC8881643; doi:10.1016/j.dcn.2022.101088)
Supplement: Supplementary file 1 — Supplementary material. [file mmc1.docx]

**SUPPLEMENTARY MATERIALS**

**Compulsive online gaming**

Compulsive online gaming was measured by self-report of the Compulsive Internet Use Scale (CIUS) (Meerkerk et al. 2009) at three time-points. The CIUS consists of 14 questions (e.g., “*How often do you continue online gaming while you intended to quit?”* and *“How often do you think you should spend less time online gaming?”*) which could be rated on a 5-point Likert scale ranging from 1 (never) to 5 (very often). The internal consistency of the CIUS has shown to be high (*α*=.90, (Meerkerk et al. 2009)). Within our sample, Cronbach’s alpha based on the 14 items was excellent for all three waves (T1: *α*=.94, T2: *α*=.95; T3: *α*=.96). Stability of compulsive online gaming within individuals over time was moderate (ICC=.70). We computed a mean score for compulsive online gaming for each individual at each of the three time-points. Higher scores indicate more compulsive online gaming.

**Sex effects compulsiveness**

On all timepoints, girls reported significantly higher levels of compulsive social media use than boys, whereas boys reported significantly higher levels of compulsive online gaming than girls on all timepoints (**Table S1, Figure S1**).

**
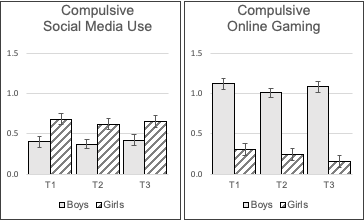
**

**Figure S1.** Sex effects for the two versions of the Compulsive Internet Use Scale for each of the timepoints separately.

**Table S1.** Age and sex effects of the two versions of the Compulsive Internet Use Scale for each of the timepoints separately.

|  |  | **Age effects** | | **Sex effects** | | | | | |
| --- | --- | --- | --- | --- | --- | --- | --- | --- | --- |
|  |  | *r* | *p-value* | *Boys (Mean/SD)* | | *Girls (Mean/SD)* | | *t-value* | *p-value* |
| Compulsive social media use ^a^ | T1 | **0.28** | **<.001** | **0.40** | **0.57** | **0.68** | **0.67** | **-2.87** | **.005** |
|  | T2 | **0.38** | **<.001** | **0.37** | **0.53** | **0.62** | **0.66** | **-2.66** | **.009** |
|  | T3 | 0.11 | .161 | **0.42** | **0.61** | **0.65** | **0.70** | **-2.34** | **.020** |
| Compulsive online gaming | T1 | **-0.20** | **0.010** | **1.121** | **0.759** | **0.307** | **0.48** | **8.226** | **<.001** |
|  | T2 | **-0.23** | **0.004** | **1.007** | **0.743** | **0.244** | **0.462** | **7.912** | **<.001** |
|  | T3 | **-0.21** | **0.006** | **1.081** | **0.762** | **0.153** | **0.371** | **10.31** | **<.001** |

^a^ *also described in Table 7*
